# Label-Free Detection of Insulin and Glucagon within Human Islets of Langerhans Using Raman Spectroscopy

**DOI:** 10.1371/journal.pone.0078148

**Published:** 2013-10-22

**Authors:** Janneke Hilderink, Cees Otto, Cees Slump, Aufried Lenferink, Marten Engelse, Clemens van Blitterswijk, Eelco de Koning, Marcel Karperien, Aart van Apeldoorn

**Affiliations:** 1 Department of Developmental BioEngineering, University of Twente, Enschede, The Netherlands; 2 Department of Medical Cell Biophysics, University of Twente, Enschede, The Netherlands; 3 Department of Systems and Signals, University of Twente, Enschede, The Netherlands; 4 Department of Nephrology, Leiden University Medical Center, Leiden, The Netherlands; 5 Department of Tissue Regeneration, University of Twente, Enschede, The Netherlands; Children's Hospital Boston, United States of America

## Abstract

Intrahepatic transplantation of donor islets of Langerhans is a promising therapy for patients with type 1 diabetes. It is of critical importance to accurately monitor islet quality before transplantation, which is currently done by standard histological methods that are performed off-line and require extensive sample preparation. As an alternative, we propose Raman spectroscopy which is a non-destructive and label-free technique that allows continuous real-time monitoring of the tissue to study biological changes as they occur. By performing Raman spectroscopic measurements on purified insulin and glucagon, we showed that the 520 cm^-1^ band assigned to disulfide bridges in insulin, and the 1552 cm^-1^ band assigned to tryptophan in glucagon are mutually exclusive and could therefore be used as indirect markers for the label-free distinction between both hormones. High-resolution hyperspectral Raman imaging for these bands showed the distribution of disulfide bridges and tryptophan at sub-micrometer scale, which correlated with the location of insulin and glucagon as revealed by conventional immunohistochemistry. As a measure for this correlation, quantitative analysis was performed comparing the Raman images with the fluorescence images, resulting in Dice coefficients (ranging between 0 and 1) of 0.36 for insulin and 0.19 for glucagon. Although the use of separate microscope systems with different spatial resolution and the use of indirect Raman markers cause some image mismatch, our findings indicate that Raman bands for disulfide bridges and tryptophan can be used as distinctive markers for the label-free detection of insulin and glucagon in human islets of Langerhans.

## Introduction

Type 1 diabetes is a chronic disease that is characterized by uncontrollable hyperglycemia, caused by the autoimmune destruction of beta-cells. This results in loss of insulin production, requiring patients to self-administer exogenous insulin daily to control blood sugar levels. Type 1 diabetes affects up to 20 million people worldwide [1]. The annual incidence rate of juvenile (age < 15) type 1 diabetes varies globally, from less than 1/100,000 in most Asian countries to 37/100,000 in certain areas of Europe, and this number is predicted to rise rapidly in the coming years [2-4]. Over time, diabetes can damage the heart, blood vessels, eyes, kidney and nerves, increasing the risk of developing life-threatening diseases such as heart disease, stroke, and kidney failure [5]. Consequently, diabetes and its complications have a significantly adverse economic impact [6]. Type 1 diabetes patients who suffer from severe hypoglycemia unawareness upon daily injections of insulin, or patients who have had a kidney transplant and take immunosuppressant drugs, benefit from intrahepatic transplantation of donor islets of Langerhans as an alternative to whole pancreas transplantation. In this procedure, purified islets extracted from donor pancreata are infused into the portal vein of the patient’s liver [7]. Although the initial clinical results are promising, only a minority (approximately 10%) of the patients were insulin-independent 5 years after treatment [8,9]. The need for ongoing immunosuppressive therapy and the scarcity of donor islets have limited its widespread clinical use.

To ensure successful transplantation, it is crucial to accurately assess critical parameters such as beta-cell mass and viability [10,11]. Conventional methods to characterize the transplant include staining with dithizone (diphenylthiocarbazone, DTZ) to determine islet purity, live/dead assays to assess cell viability, immunohistochemistry to locate insulin and glucagon, and glucose-induced-insulin-release-test (GIIST) to assess islet function. These methods are invasive, requiring sample preparation and labeling, which prohibits real-time monitoring of the tissue. Other studies have shown non-invasive in vivo monitoring of beta-cells and islets using MRI [12], bioluminescence [13] and nuclear imaging [14]. However, the spatial resolution of these methods is usually limited, which does not allow imaging at the individual cell level.

As an alternative, we propose the use of Raman spectroscopy for the non-destructive and label-free characterization of islets of Langerhans. Raman spectroscopy is an analytical technique that is based on the inelastic scattering of monochromatic light [15]. Upon interaction with the sample, a small fraction of the light (1 in 10^6^-10^8^ photons) is emitted with a different wavelength from the incident light. This wavelength shift is specific for the chemical bonds within the sample, providing a fingerprint of its entire molecular composition [16]. 

Raman spectroscopy enables characterization of living cells and tissues under physiological conditions, and is widely used in biological applications [17-20]. Rong et al. demonstrated the real-time monitoring of single rat pancreatic beta-cells using Raman microspectroscopy [21]. Moreover, Raman and infrared spectroscopy have been used for diagnosis of various diseases, including type 1 diabetes [22,23]. Using pattern-recognition methods, metabolic fingerprints of human serum samples were correlated to disease state [24]. At present, no spectroscopic measurements of human pancreatic islets have yet been reported. This study demonstrates the feasibility of using Raman microspectroscopy to characterize islets of Langerhans intended for transplantation in patients with type 1 diabetes.

## Materials and Methods

### Cell culture

MIN6-B1 mouse insulinoma cells (kindly provided by Dr. P. Halban, University Medical Center, Geneva, Switzerland) [25] were cultured in high-glucose DMEM with 2.5mM L-glutamine (Invitrogen) supplemented with 10% FBS, 100 U/ml streptomycin, 100 μg/ml penicillin and 70μM freshly added beta-mercaptoethanol. INS-1E rat insulinoma cells (kindly provided by Dr. B. Guigas, LUMC, Leiden, The Netherlands and Dr. P. Maechler, University Medical Center, Geneva, Switzerland) [26] were cultured in RPMI with 2.05 mM L-glutamine (Invitrogen) supplemented with 5% FBS, 100 U/ml streptomycin, 100 μg/ml penicillin, 10 mM HEPES, 1 mM sodium pyruvate, 50 μM freshly added beta-mercaptoethanol. AlphaTC1-6 rat alpha-cells (ATCC, Manassas, USA) were cultured in high-glucose DMEM with 2.5mM L-glutamine (Invitrogen) supplemented with 10% FBS, 100 U/ml streptomycin, 100 μg/ml penicillin, 15 mM HEPES, 1 mM sodium pyruvate, 0.1 mM non-essential amino acids, 0.02% bovine serum albumin. Cell cultures were maintained at 37°C in humidified air containing 5% carbon dioxide. Medium was refreshed every 3 to 4 days and cells were replated when 80% confluency was reached. 

### Human islet culture

Human cadaveric donor pancreata (provided by the Leiden University Medical Center, Leiden, The Netherlands) were procured through a multi-organ donor program. Tissue samples from the pancreata were used in the study if research consent was present and pancreata could not be used in clinical beta-cell therapy programs according to national laws. Islets were dispersed into single cells by adding 0.025% trypsin solution containing 10 µg/mL DNase (Pulmozyme, Genentech) and seeded onto agarose microwells for controlled cell aggregation. Intact islets and human islet cell aggregates were cultured in CMRL 1066 medium (5.5 mM glucose) (Mediatech) supplemented with 10% FCS, 20 μg/ml ciprofloxacin, 50 μg/ml gentamycin, 2 mM L-glutamin, 0.25 μg/ml fungizone, 10 mM HEPES and 1.2 mg/ml nicotinamide. Cell cultures were maintained at 37°C in a 5% CO_2_ humidified atmosphere. Medium was refreshed every 3 to 4 days. 

### Immunohistochemistry

To visualize insulin and glucagon, human islets were fixed in PBS containing 4% (w/v) paraformaldehyde, incubated overnight in 20% sucrose at 4°C, embedded in cryomatrix (Thermo Scientific), and sectioned (8 μm) using a cryotome (Shandon). Blocking was done with 0.1% (w/v) normal donkey serum/PBS for 1 hour and antibodies were diluted in 1% lamb serum/PBS. Primary antibodies used were: 1:100 rabbit-anti-glucagon (Vector Labs) incubated overnight at 4°C and 1:200 guinea pig-anti-insulin (ABCAM) incubated for 1.5 hours at 21°C. Secondary antibodies used were: 1:200 biotin donkey anti-rabbit (Jackson ImmunoResearch), 1:200 streptavidin-Alexa488 (Invitrogen), 1:400 rhodamine donkey anti-guinea pig (Jackson ImmunoResearch), all incubated for 1 hour at 21°C. Counterstaining was performed with 1 µg/mL 4'-6-diamidino-2-phenylindole (DAPI). Fluorescence was visualized using a Nikon Eclipse E600 microscope. 

### Confocal Raman microspectroscopy

UV-grade calcium fluoride (CaF_2_) slides (Crystan LTD) were used as sample substrates, because of their low background signal. Purified human recombinant insulin and synthetic glucagon (Sigma), both in powder form, were placed on CaF_2_ slides. For cells and islet preparations, CaF_2_ slides were first sterilized using 70% ethanol and coated with 0.01% poly-L-lysine (Sigma). INS-1E, alphaTC6 and MIN6 cells were seeded on coated CaF_2_ slides at 30,000 cells/cm^2^, allowed to attach overnight, fixed using 10% buffered formalin, and kept in PBS. Cryosections of human islets were placed on coated CaF_2_ slides. Subsequently, slides were washed in demiwater and air-dried. Raman spectra were acquired using a confocal Raman microspectrometer, similar to the setup described previously [27]. Briefly, a krypton ion laser (Innova 90-K, Coherent Inc.) emitting at 647.1 nm at a power of 35mW under the objective was used as the excitation source. A 63x/1.0NA water immersion objective (Zeiss W-plan Apochromat) or a 40×/0.75 NA objective (Olympus UPFLN) was used to focus the laser light over the sample. The Raman scattered photons were collected and dispersed on an air-cooled electron-multiplying charge-coupled device (EMCCD; Newton DU-970N, Andor Technology). Raman spectra were acquired in “Raman imaging mode”, in which images were obtained by stepping the laser beam over the sample in a raster pattern, while collecting complete spectral information during each step. Average Raman fingerprints of the single cells were obtained by scanning a 32x32 raster pattern over a sample area of 20x20 µm with a step size of 0.6 µm and an accumulation time of 0.5 s/step, resulting in datasets consisting of 1024 spectra per 400 µm^2^ encompassing a single cell. Three-level cluster analysis was performed to separate the spectra corresponding to the cytoplasm and the spectra corresponding to the cell nucleus from the background spectra. Spectral data from 5 randomly chosen cells was averaged. Raman imaging on beta-cells was performed by scanning a 64x64 raster pattern over a sample area of 20x20 µm with a step size of 0.3 µm, and an accumulation time of 1s/step. Raman imaging on human islet sections was performed by making 16 (4x4) subsequent scans, each consisting of a 16x16 raster pattern over an area of 30x30 µm with a step size of 1.88 µm and an accumulation time of 0.5s/step. The scans were made exactly 30 µm from each other by using a Kleindiek LT6820 microscope substage controlled by a Kleindiek NanoControl (Kleindiek Nanotechnik, Reutlingen, Germany). The spectral data of the scans were then combined (using MATLAB 7.6, The MathWorks) in one large data matrix consisting of 4096 spectra, covering a total sample area of 120x120 µm. High-resolution Raman imaging on subparts of the islet preparations were performed by scanning a 32x32 raster pattern over a sample area of 30x30 µm with a step size of 0.94 µm and an accumulation time of 1s/step.

### Raman data analysis

Raman spectra were preprocessed, as described previously [28,29], by: (1) removing cosmic ray events; (2) subtracting camera offset; (3) calibration of the wave number axis; and (4) correction of frequency-dependent transmission. The well-known band-positions of toluene were used to relate wavenumbers to pixels [30]. The frequency-dependent optical detection efﬁciency of the setup was corrected using a tungsten halogen light source (Avalight-HAL; Avantes BV) with a known emission spectrum. The detector-induced etaloning effect was also compensated by this procedure. Noise in the 3D (spatial x spatial x spectral dimension) data matrix was reduced by singular value decomposition. Univariate and multivariate analyses were performed over the hyperspectral Raman data as described previously [28,31]. Univariate analysis was performed by a selection of specific vibrational bands of interest and an integration of the band intensity after local baseline subtraction. Multivariate analysis, involving both principal component analysis (PCA) and hierarchical cluster analysis (HCA) using Ward’s clustering method, was performed on Raman imaging data matrices to visualize regions in cells with high Raman spectral similarities [32,33]. Data for PCA was preprocessed by auto-scaling to the mean of the spectra along the frequency axis. HCA makes use of the scores obtained from the PCA data and was preprocessed with a first derivative Savitsky-Golay algorithm (with seven-point smoothing and quadratic polynomial fit) to minimize small variations in the baseline, and subsequently auto-scaled to the mean of the spectra along the frequency axis. For quantitative comparison between Raman and fluorescence imaging, the Dice coefficient [34] was calculated as follows: 2*h*/*(a*+*b*), in which h is the overlap between the two (binary) images, and *a* and *b* represent the surface areas in the separate images. The Dice coefficient ranges from 0 to 1, in which 0 indicates no overlap and 1 indicates perfect agreement. All data manipulations were performed in routines written in MATLAB 7.6 (The MathWorks). HCA and PCA were performed using PLS toolbox (Eigenvectors Research Inc.).

## Results

### Raman spectra of purified hormones

**Figure 1:**
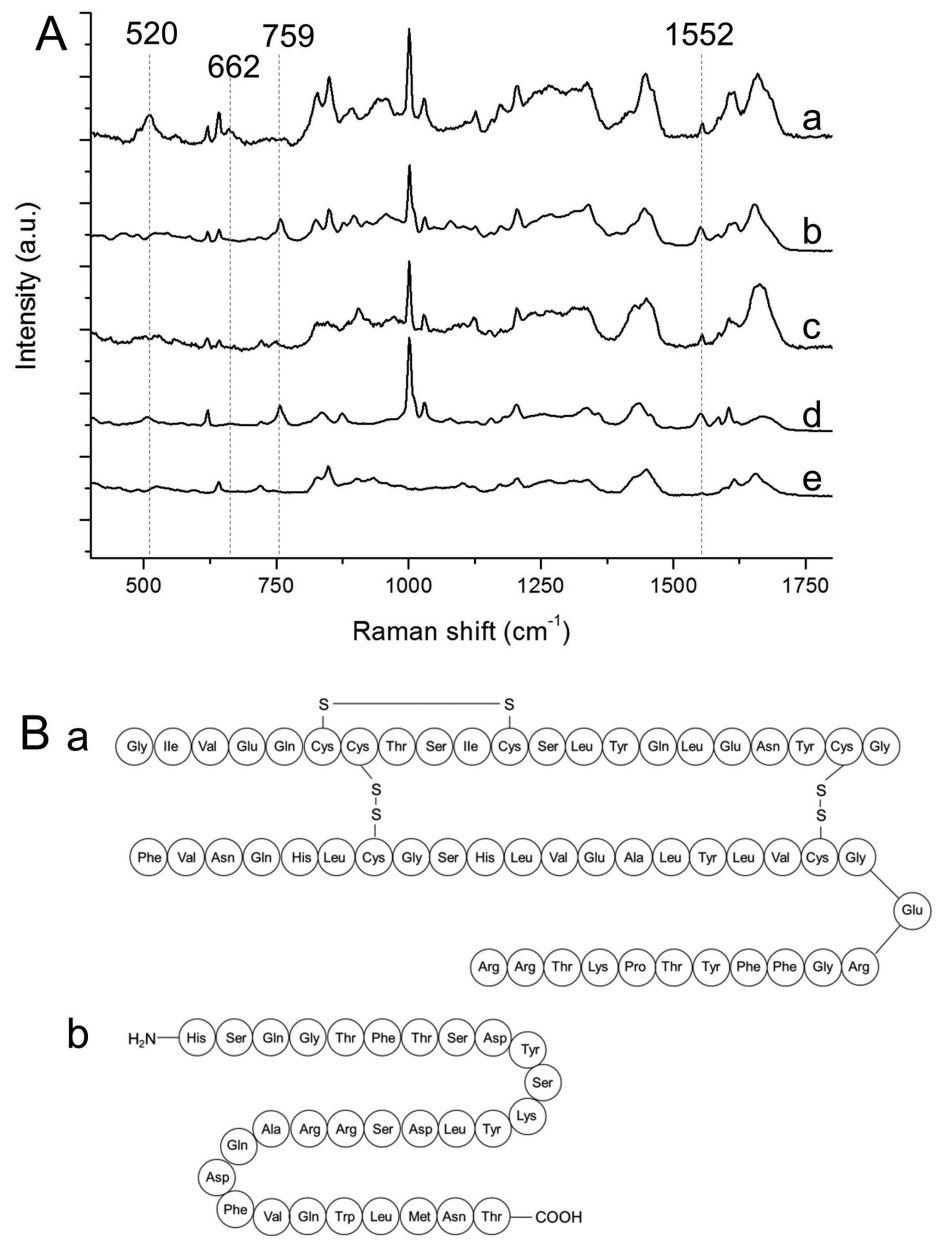
Confocal Raman spectroscopy of purified hormones. (A) Raman fingerprint of insulin (a), glucagon (b), amylin (c), somatostatin (d), pancreatic polypeptide (e). Spectra are vertically offset for clear representation. Bands specific for sulfide bridges between cysteines in insulin were found at 520 and 662 cm-1. Tryptophan-specific bands at 759 and 1552 cm-1 were found in spectrum of glucagon. (B) Amino acid composition of insulin (a), containing three Raman-active sulfide bridges between cysteine groups, and glucagon (b), containing the Raman-active amino acid tryptophan.

Raman spectroscopy on purified hormones was performed to identify Raman markers for insulin and glucagon, the two main hormones within the islets of Langerhans produced by the beta-cells (50-70%) and alpha-cells (20-35% of islet cells), respectively [35,36]. Spectra a and b in Figure 1A represent the Raman spectra of insulin and glucagon. Similarities in the spectra are mainly caused by vibrations in phenylalanine and tyrosine, amino acids that are part of the molecular structure of both insulin and glucagon. More interestingly, spectral differences between the two hormones were observed that could be explained by their molecular structure (Figure 1B). Insulin contains six cysteine groups that are linked with three disulfide (S-S) bridges. Molecular vibrations of these bridges result in specific Raman bands at 520 cm^-1^ (S-S vibration) and 662 cm^-1^ (C-S stretching) [37-39]. Tryptophan, one of the building blocks of glucagon, has molecular vibrations that result in Raman bands at 759 (aromatic ring breathing) and 1552 cm^-1^ (C=C vibration) [37]. Because tryptophan is not present in insulin and disulfide bridges are not present in glucagon, these Raman bands may be used to distinguish between insulin and glucagon. However, since both tryptophan and disulfide bridges between cysteines are also present in other polypeptides, these amino acids may only serve as indirect markers of glucagon and insulin.

Additionally, we measured a number of other hormones that are produced by islets of Langerhans, albeit in much lower amounts than insulin and glucagon. Amylin is co-secreted with insulin by beta-cells (molar ratio amylin to insulin ~2:100) [40], somatostatin is produced by delta-cells (<10% of total islet cells), and pancreatic polypeptide is produced by PP-cells (<5% of cells) [36]. Pancreatic polypeptide does not contain any tryptophan or cysteine groups, reflected by the absence of the corresponding Raman bands. The polypeptides amylin and somatostatin both contain one disulfide bridge, which in contrast to the three bridges in insulin, results in strongly reduced bands at 520 and 662 cm^-1^. Like glucagon, somatostatin contains one amino acid tryptophan and thus also shows Raman bands at 759 and 1552 cm^-1^ (Figure 1A). 

### Raman fingerprint of alpha- and beta-cell lines

**Figure 2:**
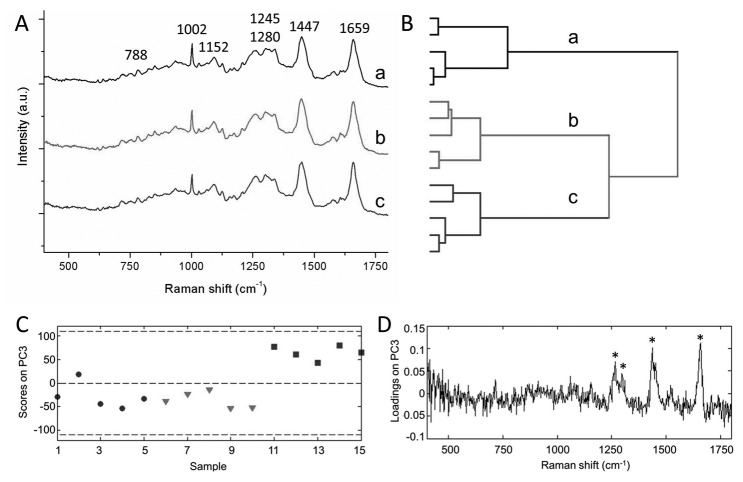
Raman spectroscopic evaluation of insulinoma cell lines. (A) Average Raman fingerprints of MIN6 (a), INS-1E (b) and alpha-TC6 (c) cells were acquired by averaging the spectra of 5 randomly chosen individual cells. Spectra are vertically offset for clear representation. (B) Multivariate data analysis was applied to the dataset to objectively assess subtle differences in the fingerprint region of the spectra. Hierarchical cluster analysis (HCA) classifies the various cell types in separate clusters. (C) PC3 scores from PCA on spectra of the cell cytoplasm clearly distinguish the alpha from the beta cells. Black circles: MIN6 cells, red triangles: INS-1E cells, blue squares: alpha-TC6 cells. (D) PC3 loadings indicate the Raman bands that are mainly involved in the separation of alpha and beta cells. Band positions characteristic for unsaturated fatty acids are marked with an asterisk.

Raman spectral mapping was performed on INS-1E rat insulinoma beta-cells, MIN6 mouse insulinoma beta-cells and alphaTC6 rat alpha-cells. The averaged spectra (n=5) for each cell type are shown in Figure 2A. The spectra show several bands characteristic for cells and biological samples, which can be assigned to DNA (788 cm^-1^), phenylalanine (1002 cm^-1^), proteins (1152 cm^-1^), lipids and amides (1245-1280 cm^-1^), CH_2_ bending of proteins and lipids (1447 cm^-1^), and C=C groups in unsaturated fatty acids (1659 cm^-1^). HCA on the averaged total cell spectra successfully classifies the spectra in three separate clusters. Although there is some variation within one cell type, the variance between the spectra of one cell type is much smaller than the variance between the spectra of the different cell types (Figure 2B). 

In addition to the total cell spectra, we have investigated the molecular fingerprint of just the cytoplasm of the cell, where proteins are stored in the secretory vesicles. A three-level cluster analysis was performed to separate the spectra corresponding to the cytoplasm from the spectra corresponding to the nucleus or background. Principal component analysis (PCA) was performed for unsupervised analysis of the cytoplasm spectra. This method assigns scores to reveal subsequent levels of variation in the dataset, with the first principal component (PC) reflecting the highest amount of variation. Each spectrum in the dataset acquires a score with respect to each level of variation. Following the analysis it was observed that the third principal component (responsible for 1.4% of the variance) accounts for the separation of the alpha-cell line from the beta-cell lines. PC1 (91.5%) and PC2 (6.1%) did not contribute to distinguish the various cell types, and represent intercellular variation. Figure 2C shows the individual scores of PC3 for each Raman spectrum analyzed, in which two distinct groups were observed. The INS-1E and MIN6 beta-cells are characterized by negative PC3 scores, while the alphaTC6 alpha-cells show positive PC3 scores. To determine the biochemical features that are responsible for clustering of the two groups, the loadings on PC3 were analyzed (Figure 2D). The PC3 loadings spectrum shows bands at 1264, 1298, 1440 and 1660 cm^-1^, which resemble lipid contributions of predominantly unsaturated fatty acids. In addition, the band at 716 cm^-1^ is characteristic for the phospholipid phosphatidylcholine. This indicates that differences in intracellular lipid concentration account for the classification of (alphaTC6) alpha-cells and (MIN6 and INS-1E) beta-cells.

### Raman imaging of INS-1E beta-cells

**Figure 3:**
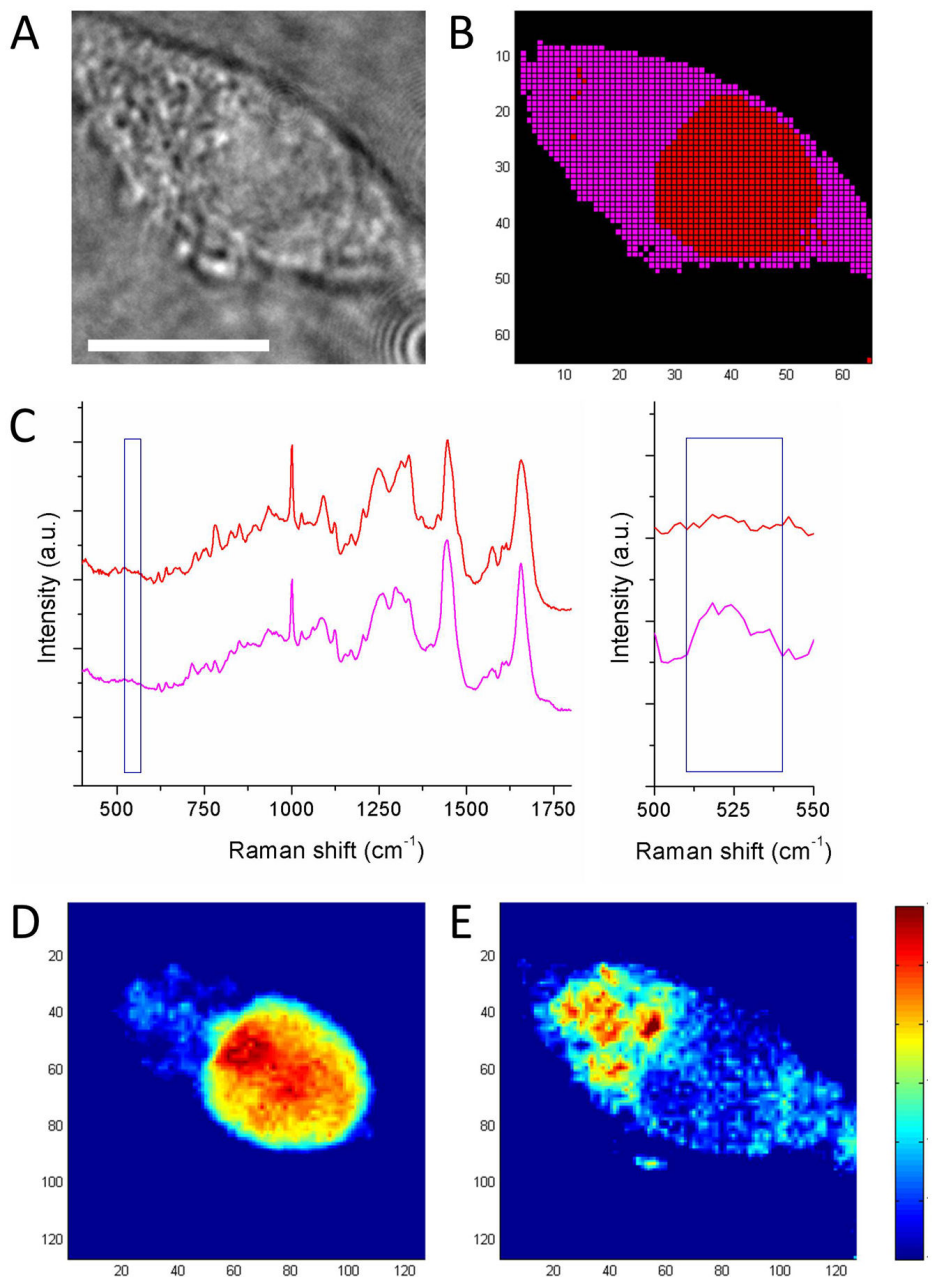
Label-free Raman imaging of rat insulinoma INS-1E beta-cells. (A) Brightfield image of a beta-cell. (B) Raman cluster image acquired by scanning the confocal laser beam in a 64x64 raster pattern. Analysis with 3 levels of clustering illustrates the spatial variation of Raman signal within a cell by distinguishing subcellular structures. (C) Corresponding Raman cluster spectra (corrected for background) show the average signal for cytoplasm (magenta) and nucleus (red). Enlargement shows the Raman band at 520 cm-1, specific for difsulfide-bridged cysteine. Spectra are vertically offset for clear representation. Integrating over specific Raman bands shows (D) the distribution of DNA (783 cm-1, Δ = 26 cm-1) corresponding to red cluster in B, and (E) the distribution of disulfide bridges between cysteine groups (524 cm-1, Δ = 30 cm-1). Scale bar represents 10 µm.

High-resolution Raman imaging is performed on individual INS-1E cells to identify intracellular insulin. INS-1E cell morphology was examined using bright field microscopy and the same cell was imaged using Raman microspectroscopic scanning (Figure 3A). Three-level hierarchical cluster analysis of the dataset was performed, resulting in the Raman cluster image (Figure 3B). This illustrates the spatial variation of the Raman signal by distinguishing the nucleus (red cluster) and cytoplasm of the cell (purple cluster) from the background (black cluster). Figure 3C shows the corresponding cluster spectra for nucleus and cytoplasm, corrected for the background spectrum. Univariate analysis on selected Raman bands was performed to visualize the presence and distribution of specific compounds. The intensity of the 788 cm^-1^ band for DNA was used to locate the cell nucleus (Figure 3D), which corresponds to the red cluster in Figure 3B. Raman mapping of the 520 cm^-1^ band shows high local concentrations of disulfide-bridged cysteine groups which could indicate the accumulation of insulin in the cytoplasm of the INS-1E cell at these locations (Figure 3E). 

### Raman imaging of human pancreatic islets

**Figure 4:**
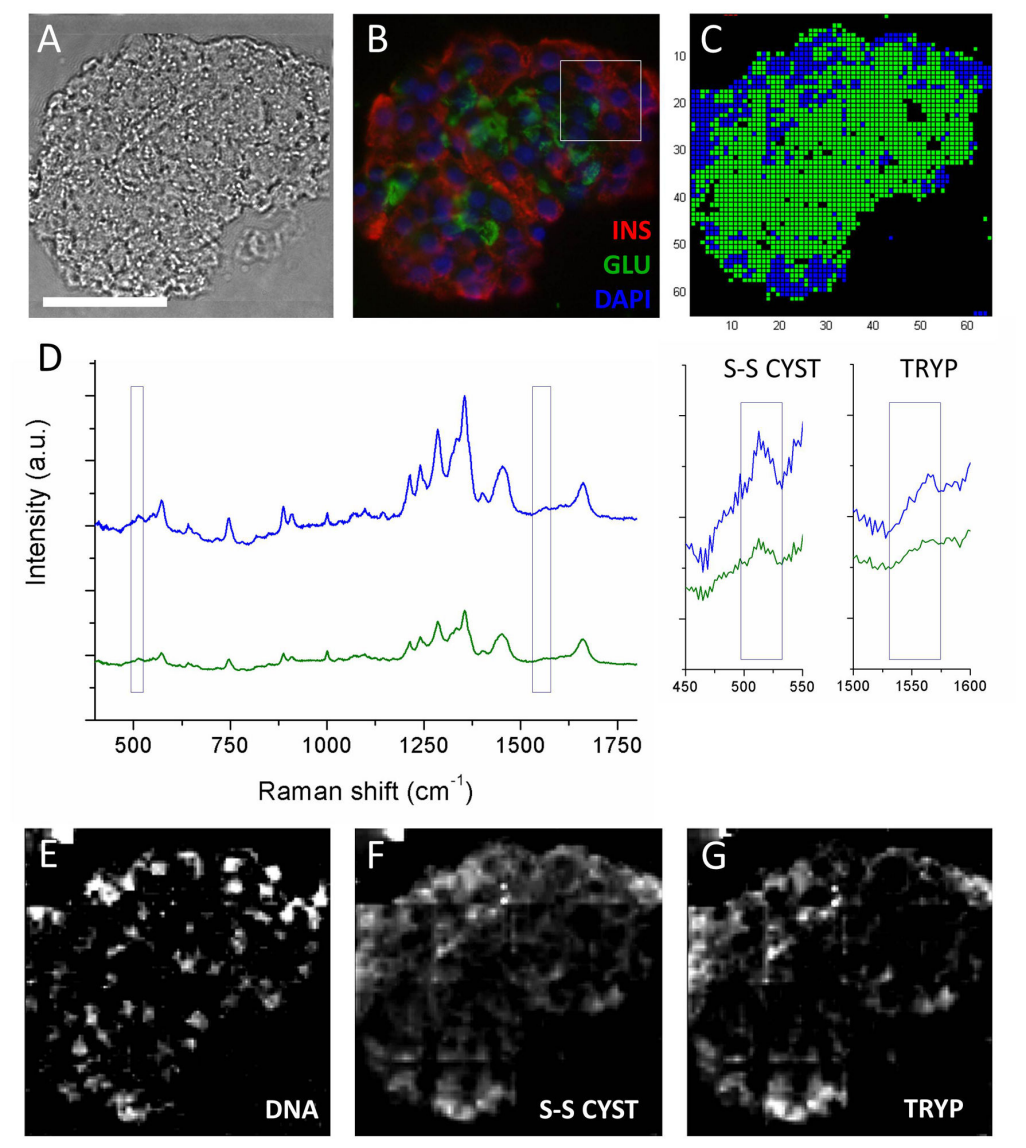
Label-free Raman imaging of primary human islets of Langerhans. (A) Brightfield microscopy image of a cryosection of an islet of Langerhans. (B) Fluorescence microscopy image of the islet stained for DNA (blue), insulin (red) and glucagon (green). (C) Corresponding Raman cluster image and (D) corresponding Raman cluster spectra, acquired by scanning the confocal laser bundle in a 64x64 raster pattern. Analysis with 3 levels of clustering illustrates the spatial variation of Raman signal within the islet. Enlargement shows the disulfide band at 520 and the tryptophan band at 1552 cm-1. Spectra are vertically offset for clear representation. The bottom panel shows hyperspectral Raman images for (E) DNA (783 cm-1, Δ = 26 cm-1), (F) disulfide bridges between cysteine groups (524 cm-1, Δ = 30 cm-1) and (G) tryptophan (1545 cm-1, Δ = 21 cm-1). Minor artifacts caused by the image-stitching procedure are visible in the Raman images. Scale bar represents 50 µm.

We have performed hyperspectral Raman imaging (with a step size of 1.88 µm) on human donor islets and compared their spectral characteristics to those of pure insulin and glucagon. A brightfield microscopy image of a representative human islet section is shown in Figure 4A. The islets were stained for insulin and glucagon to visualize the distribution of alpha- and beta-cells (Figure 4B). Three-level cluster analysis was performed on the entire fingerprint region to objectively assess the spatial variation of the Raman signal. The green and blue clusters, representing the islet, indicate some variation in the concentration of DNA, lipids and proteins throughout the tissue (Figure 4C and D). Univariate Raman spectral imaging was performed to investigate the distribution of specific compounds within the whole islet section. The intensity and distribution of the 788 cm^-1^ band visualizes the presence of DNA, and was used to identify the cell nuclei in islet cells. Moreover, the distribution of the Raman band at 520 cm^-1^ specific for disulfide bridges between cysteine groups, and the tryptophan band at 1552 cm^-1^ are depicted in Figure 4E-G. However, at low resolution these bands did not clearly correlate with the immunolabeling against insulin and glucagon.

### High resolution Raman imaging of human pancreatic islets

**Figure 5:**
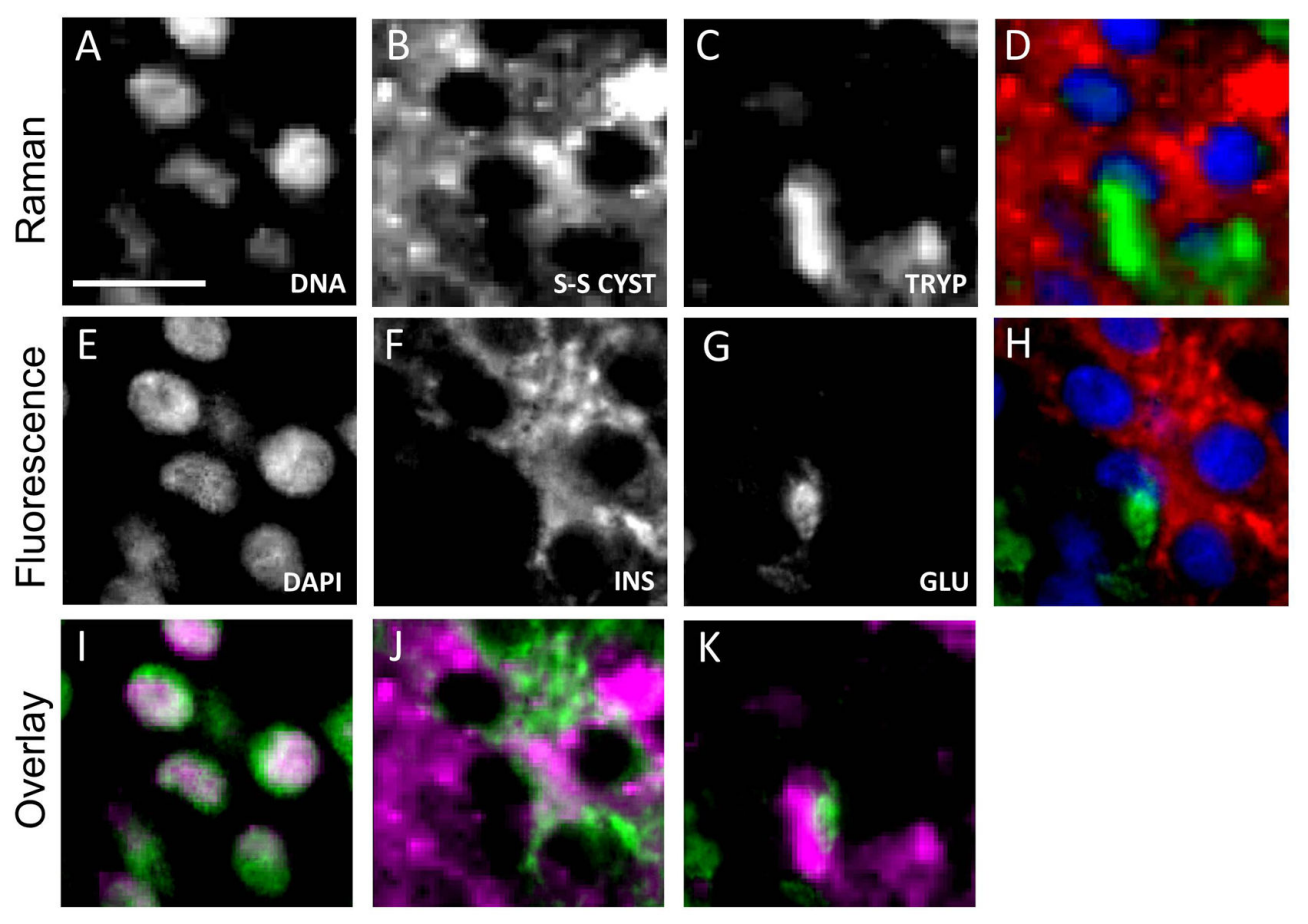
Label-free detection of insulin and glucagon in human islets. The top panel shows the univariate Raman images of a cryosection of an islet of Langerhans for (A) DNA (783 cm-1, Δ = 26 cm-1), (B) disulfide bridges between cysteine groups (524 cm-1, Δ = 30 cm-1) and (C) tryptophan (1545 cm-1, Δ = 21 cm-1). The second panel shows the corresponding fluorescence microscopy images stained for (E) DNA, (F) insulin and (G) glucagon. The bottom panel shows the overlay images of the Raman image (in purple) and fluorescence image (green) for (I) DNA, (J) insulin and (K) glucagon. (D) Merged Raman spectroscopy image images of DNA (blue), difsulfide bridged cysteine (red) and tryptophan (green). (H) Merged fluorescence image of DAPI (blue), insulin (red) and glucagon (green). Scale bar represents 50 µm.

Since proteins are stored in small secretory vesicles in the cell cytoplasm, we performed high-resolution hyperspectral Raman imaging with a step size of 0.94 µm on part of the islet preparation (as indicated by a white square in Figure 4B). Figure 5A-C visualizes the distribution of Raman bands specific for DNA, disulfide bridges in cysteine and tryptophan. At this higher (subcellular) resolution, a clear distribution of these molecules was observed which coincided with fluorescence labeling for cell nuclei, insulin and glucagon, respectively (Figure 5E-G). To compare the obtained Raman images with images obtained using conventional fluorescence microscopy, we generated overlay images with the Raman signal in purple and the fluorescence signal in green (Figure 5I-K). A quantitative comparison was made by calculating the Dice coefficient, which gives a value for overlap between the two images. For DNA, we obtained a Dice coefficient of 0.69 indicating a strong correlation between the Raman signal and the fluorescence signal, which is depicted in the corresponding overlay image. We also found a clear correlation for insulin and glucagon (Figure 5J and K). However, this correlation is somewhat lower than for DNA resulting in Dice coefficients of 0.36 for insulin and 0.19 for glucagon. The merged Raman image shows the distribution of DNA, disulfide bridges in cysteine and tryptophan (Figure 5D), whereas Figure 5H represents the merged image obtained by conventional fluorescence immunolabeling. 

## Discussion

Our study has explored the feasibility of Raman spectroscopy to identify insulin and glucagon within human islets of Langerhans. We used Raman spectroscopy to characterize cell lines and human donor pancreatic islets at the sub-cellular level. 

By performing Raman measurements on purified hormones we showed that Raman bands specific for disulfide bridges between cysteine groups in insulin and tryptophan in glucagon are mutually exclusive, and could therefore be used for the label-free distinction between both hormones. High-resolution univariate Raman imaging for the 520 cm^-1^ band on individual beta-cells showed the distribution of disulfide bridges in the cytoplasm of the cells. These findings correlate with the knowledge that insulin is accumulated in large dense-core vesicles within the cytoplasm of pancreatic beta-cells and therefore locally contain high concentrations of cysteine [41,42], suggesting that Raman spectroscopy allows for the label-free detection of intracellular insulin.

Subsequently, we investigated whether we could use the Raman markers specific for disulfide bridges and tryptophan to distinguish between insulin-producing beta-cells and glucagon-producing alpha-cells within human islets of Langerhans. Hyperspectral imaging on human islet preparations with a step size of 1.88 µm enabled identification of individual cells but did not provide detailed subcellular information. Since hormones such as insulin and glucagon are stored in vesicles with an estimated diameter of 0.3 µm [43], high-resolution hyperspectral Raman imaging was performed on smaller areas of the islet preparation in which we decreased the step size from 1.88 to 0.94 µm, resulting in more detailed information. In this case every pixel represents 0.88 µm^2^ of the sample, providing molecular information on a sub-micrometer level. Univariate analysis of selected Raman bands was performed to display the distribution of DNA, disulfide-bridged cysteines and tryptophan which corresponded with the corresponding fluorescence microscopy images for DNA, insulin and glucagon as demonstrated by overlaying the images obtained by both techniques. We quantitatively compared the correlation between label-free Raman imaging and conventional fluorescence microscopy by calculating the Dice coefficients (DC). The highest Dice coefficient was found for DNA (DC=0.69), while significantly lower coefficients for insulin (DC=0.36) and glucagon (DC=0.19) were determined. Several factors can be attributed to the fact that the images of both techniques do not perfectly match.

First, because tryptophan and disulfide bridges between cysteines are also present in other polypeptides, these amino acids only serve as indirect markers of glucagon and insulin. However, since both hormones are stored at high concentrations in secretory granules of alpha- and beta-cells, this should locally result in strong Raman bands at the described wavenumbers. Since insulin contains six disulfide-bridged cysteine groups, whereas glucagon only contains one tryptophan molecule, we can assume that tryptophan as a marker for glucagon is less specific than disulfide bridges are for insulin. In addition, somatostatin produced by delta cells also contains one tryptophan molecule. Delta cells which commonly comprise 11% of the islet cell population, whereas 35% is composed of alpha-cells [35], will therefore contribute to the tryptophan-specific Raman signal. 

Second, there is a considerable difference regarding the spatial resolution of both imaging methods used. Raman spectroscopic imaging is performed by stepwise moving the laser beam over the sample in a raster pattern, while simultaneously taking a single Raman spectrum at each stop, creating an array of pixels. Subsequently, band integrations are performed on bands of interest and with these data an image is reconstructed. The spatial resolution of the Raman image is thus determined by the step size of the laser focal spot. For detailed Raman imaging on islets, we used a step size of 0.94 µm. In comparison, the resolution of fluorescence microscopy is limited by the diffraction of light to a few hundred nanometers. Last, the same islet section was first imaged using Raman spectroscopy and subsequently immunolabeled for fluorescence microscopy, for which two separate microscope systems were used. 

Our findings show that high-resolution hyperspectral Raman imaging with a step size smaller than 1 µm, enables the label-free distinction of insulin and glucagon within human islets of Langerhans. However, it does not yet provide information on the viability of the tissue or determines the islets’ ability to respond to a glucose stimulus. On the other hand, Notingher and colleagues have already reported the use of Raman spectroscopy to distinguish viable from dead cells [44]. Their findings suggest the possibility of using Raman spectroscopy to quantify viability of pancreatic islet grafts. Further studies need to be performed to investigate the feasibility of performing real-time studies on viability and functioning of islet preparations using confocal Raman spectroscopy. In such analysis, it will also be beneficial to correlate the islet parameters to the final patient outcome after transplantation. This would help selecting optimal tissue for transplantation [45,46]. 

## Conclusion

We demonstrate, in contrast to conventional histological methods, that Raman spectroscopy enables the label-free classification of various cell types in human islets and pancreatic cell lines. Measurements on purified hormones revealed Raman markers specific for disulfide bridges between cysteine groups (520 cm^-1^) and tryptophan (1552 cm^-1^) that can be used to distinguish between insulin and glucagon. High-resolution Raman imaging, using sub-micrometer steps, for these specific bands enables local detection of high concentrations of insulin and glucagon in human pancreatic islet cells. This method provides information on composition and structural organization of pancreatic endocrine tissue in a label-free manner, and may help to accurately monitor islet quality before transplantation in patients suffering from type 1 diabetes in the future. 
